# COVID-19 and race/color disparity: a brief analysis of the indigenous population in a state in the Brazilian Amazon

**DOI:** 10.31744/einstein_journal/2021CE6734

**Published:** 2021-12-03

**Authors:** Arthur Arantes da Cunha, Rodolfo Antonio Corona, Emerson Augusto Castilho-Martins

**Affiliations:** 1 Universidade Federal do Amapá Macapá AP Brazil Universidade Federal do Amapá, Macapá, AP, Brazil.

Dear Editor,

After more than one year of the first case of the coronavirus disease 2019 (COVID-19) caused by severe acute respiratory syndrome coronavirus 2 (SARS-CoV-2), in Brazil,^(1)^ race/color disparity among affected individuals has great epidemiological relevance in the country.^(2)^ In this context, indigenous individuals have shown significant vulnerability to SARS-CoV-2.^(2)^ A seroprevalence survey for SARS-CoV-2 showed prevalence among natives of 6.4%, which is much higher than among whites (1.4%).^(2)^ In the United States, an expressive unequal relation has also been described among American Indians and Alaska Natives, when compared with the non-Hispanic white population.^(3)^

Although there are studies on the vulnerability of minority groups,^(2,4)^ few studies on COVID-19 have been conducted exclusively among Brazilian indigenous. Therefore, we used data from the State Department of Health of Amapá^(5)^ and from the Brazilian Institute of Geography and Statistics Automatic Retrieval System,^(6)^ to perform an ecological analysis of the occurrence of COVID-19 in the indigenous and non-indigenous population of Amapá (1°16’50.1”N 51°52’58.6”W). Amapá, a Brazilian state with a population of approximately 860 thousand inhabitants, marked by a history of low socioeconomic development, and located on the left bank of the Amazon River, is one of the regions most affected by SARS-CoV-2 in Brazil.^(2,7)^

Excluding the cases in which race/skin color of the individual was ignored, between March 20, 2020 and April 29, 2021, a total of 72,913 cases of COVID-19 were recorded in Amapá. Of this total, 4,511 (6.19%) were indigenous and 68,402 (93.81%) were non-indigenous.^(5)^ Considering the distribution according to race/color of the population,^(6)^ the number of cases reported among indigenous people was higher than among non-indigenous people, given the expected frequencies (χ^2^=1,7120.4; df=1; p value=0.0001). Furthermore, the cumulative incidence of COVID-19 in indigenous people was approximately 5.6-fold higher than among non-indigenous people (Table 1).

**Table 1:** Number of cases and cumulative incidences of COVID-19 among indigenous and non-indigenous people. State of Amapá, Brazilian Amazon, March 20, 2020 to April 29, 2021

Variable	Observed frequency* (expected^†^)	χ^2^ (p value)^‡^
Cases of COVID-19		
Indigenous	4,511 (809)	1,7120.4 (0.0001)
Non-indigenous	68,402 (72,104)	
Incidence accumulated during the period	Per 10,000 residents	Difference %
Indigenous	4,481.9	458.1
Non-indigenous	803.1	
Population of the state of Amapá	Distribution %	
Indigenous	1.11	
Non-indigenous	98.89	

Sources: Amapá. Governo do Estado do Amapá. Secretaria de Estado da Saúde do Amapá (SESA). Painel Coronavírus. Macapá: SESA; 2021 [citado 2021 Maio 10]. Disponível em: http://painel.corona.ap.gov.br/;^(5)^ Instituto Brasileiro de Geografia e Estatística (IBGE). Sistema IBGE de Recuperação Automática (SIDRA). Rio de Janeiro: SIDRA; 2021 [citado 2021 Maio 10]. Disponível em: https://sidra.ibge.gov.br/Tabela/3175;^(6)^ Amapá. Governo do Estado do Amapá. Secretaria Extraordinária dos Povos Indígenasn (SEPI). Macapá: SEPI; 2021 [citado 2021 Maio 10]. Disponível em: http://www.sepi.ap.gov.br/interno.php?dm=961.^(8)^

* Quantity of registered COVID-19 cases that had ‘race/skin color’ record; ^†^ Expected frequency of compliance χ^2^ test, in reference to the proportion of the population of the state of Amapá; ^‡^ compliance χ^2^ test.

This discrepancy in COVID-19 infection between indigenous and non-indigenous people in Amapá is possibly due to the susceptibility of indigenous people to SARS-CoV-2, which may be mediated by socioeconomic, sociodemographic, and/or genetic factors.^(2-4)^ Thus, ethnic minorities in contexts of low social development, as is the situation of much of the population of Amapá, may have a greater chance of infection, as well as of developing more severe cases of COVID-19.^(4,7)^

In Amapá, approximately 20% of indigenous people live in urban areas (https://indigenas.ibge.gov.br/estudos-especiais-3.html), and large parts of this indigenous population residing in cities, as well as an important part of the general population of the state, live in subnormal agglomerations, with low *per capita* income, and lack of sanitation and medical-hospital services.^(7)^ Regarding indigenous villagers, it should be noted that in Brazil, even during the pandemic, illegal miners and loggers continued to operate on indigenous lands, which might have increased the possibility of exposure to SARS-CoV-2 and the occurrence of outbreaks of the disease.^(9)^

Therefore, there is a great disparity in the occurrence of COVID-19 between indigenous and non-indigenous people in the state of Amapá. It is suggested that individual studies be conducted to investigate this relation of vulnerability. Furthermore, it is necessary that government authorities improve the support measures for the indigenous population of the state, with more extensive vaccination and social and health support for indigenous people, regardless of their place of residence.
