# Supplementary material for: COVID-19 and race/color disparity: a brief analysis of the indigenous population in a state in the Brazilian Amazon
Source: Einstein (Sao Paulo). 2021 Dec 3;19:eCE6734. doi: 10.31744/einstein_journal/2021CE6734 (PMC8664283; doi:10.31744/einstein_journal/2021CE6734)
Supplement: Translation

# Translation: COVID-19 e as disparidades segundo raça/cor: uma breve análise acerca da população indígena em um estado da Amazônia brasileira

**Authors:** Arthur Arantes da Cunha, Rodolfo Antonio Corona, Emerson Augusto Castilho-Martins

**Affiliations:** 1 Brasil Universidade Federal do Amapá, Macapá, AP, Brasil.

Caro Editor,

Após mais de 1 ano do primeiro caso da doença pelo coronavírus 2019 (COVID-19), causada pelo coronavírus da síndrome respiratória aguda grave 2 (SARS-CoV-2), no Brasil,^(1)^ as disparidades segundo a raça/cor dos indivíduos acometidos apresentam grande relevância epidemiológica no país.^(2)^ Nesse contexto, os indígenas apresentaram importante vulnerabilidade ao SARS-CoV-2.^(2)^ Um inquérito de soroprevalência para SARS-CoV-2 demonstrou prevalência entre indígenas de 6,4%, ou seja, muito superior à prevalência entre brancos (1,4%).^(2)^ Nos Estados Unidos, relação desigual expressiva também foi descrita entre indígenas americanos e nativos do Alasca, quando comparados à população branca não hispânica.^(3)^

Embora existam estudos acerca da vulnerabilidade de grupos minoritários,^(2,4)^ foram realizadas poucas pesquisas acerca da COVID-19 exclusivamente entre indígenas brasileiros. Nesse sentido, utilizamos dados da Secretaria de Estado da Saúde do Amapá^(5)^ e do Sistema do Instituto Brasileiro de Geografia e Estatística de Recuperação Automática,^(6)^ para realizar uma análise ecológica do acometimento pela COVID-19 da população indígena e da população não indígena no Amapá (1°16’50.1”N 51°52’58.6”W). O Amapá é um estado brasileiro com população de aproximadamente 860 mil habitantes, marcado por um histórico de baixo desenvolvimento socioeconômico, e localizado à margem esquerda do rio Amazonas, sendo uma das regiões mais acometidas pelo SARS-CoV-2 no Brasil.^(2,7)^

Dessa forma, excluindo-se os casos em que raça/cor do indivíduo foi ignorada, registraram-se 72.913 casos de COVID-19, entre 20 de março de 2020 e 29 de abril de 2021 no Amapá. Desse total, 4.511 (6,19%) eram indígenas e 68.402 (93,81%) não indígenas.^(5)^ Considerando-se a distribuição segundo raça/cor da população,^(6)^ o número de casos registrados entre indígenas foi maior que entre não indígenas, dadas as frequências esperadas (χ^2^=1.7120,4; df=1; valor de p=0,0001). Ademais, a incidência acumulada de COVID-19 em indígenas foi aproximadamente 5,6 vezes maior que entre não indígenas (Tabela 1).

**Tabela 1:** Quantitativo de casos e incidências acumuladas de COVID-19 entre indígenas e não indígenas. Estado do Amapá, Amazônia brasileira, 20 de março de 2020 a 29 de abril de 2021

Variável	Frequência observada* (esperada^†^)	χ^2^ (Valor de p^)‡^
Casos de COVID-19		
Indígenas	4.511 (809)	1.7120,4 (0,0001)
Não indígenas	68.402 (72.104)	
Incidência acumulada no período	Por 10.000 residentes	Diferença %
Indígenas	4.481,9	458,1
Não indígenas	803,1	
População do estado do Amapá	Distribuição %	
Indígenas	1,11	
Não indígenas	98,89	

Fontes: Amapá. Governo do Estado do Amapá. Secretaria de Estado da Saúde do Amapá (SESA). Painel Coronavírus. Macapá: SESA; 2021 [citado 2021 Maio 10]. Disponível em: http://painel.corona.ap.gov.br/;^(5)^ Instituto Brasileiro de Geografia e Estatística (IBGE). Sistema IBGE de Recuperação Automática (SIDRA). Rio de Janeiro: SIDRA; 2021 [citado 2021 Maio 10]. Disponível em: https://sidra.ibge.gov.br/Tabela/3175;^(6)^ Amapá. Governo do Estado do Amapá. Secretaria Extraordinária dos Povos Indígenasn (SEPI). Macapá: SEPI; 2021 [citado 2021 Maio 10]. Disponível em: http://www.sepi.ap.gov.br/interno.php?dm=961.^(8)^

* Quantitativo de casos de COVID-19 registrado que possuíam notificação de ‘raça/cor’; ^†^ frequência esperada do teste χ^2^ de aderência, com referência na proporção da população do estado do Amapá; ^‡^ teste χ^2^ de aderência.

Essa discrepância no acometimento pela COVID-19 entre indígenas e não indígenas no Amapá é, possivelmente, devida a uma suscetibilidade dos indígenas ao SARS-CoV-2, que pode ser mediada por fatores socioeconômicos, sociodemográficos e/ou genéticos.^(2-4)^ Dessa forma, destaca-se que minorias étnicas, em contextos de baixo desenvolvimento social, como é a situação de grande parte da população do Amapá, podem apresentar maior chance de infecção, assim como de desenvolver quadros mais graves da COVID-19.^(4,7)^

No Amapá, aproximadamente 20% dos indígenas residem em zonas urbanas (https://indigenas.ibge.gov.br/estudos-especiais-3.html), sendo que grande parte dessa população indígena residente nas cidades, assim como importante parcela da população geral do estado vive em aglomerados subnormais de habitação, com baixa renda *per capita* e carência de serviços sanitários e médico-hospitalares.^(7)^ Quanto aos indígenas aldeados, ressalta-se que, no Brasil, mesmo durante a pandemia, garimpos e madeireiros ilegais continuaram a atuar em terras indígenas, o que pode ter aumentado a possibilidade de exposição ao SARS-CoV-2 e a ocorrência de surtos da doença.^(9)^

Portanto, é grande a disparidade no acometimento pela COVID-19 entre indígenas e não indígenas no estado do Amapá. Sugere-se a elaboração de estudos individuados que investiguem essa relação de vulnerabilidade. Ademais, é necessário que as autoridades governamentais aprimorem as medidas de suporte à população indígena do estado, com maior amplitude da vacinação e apoio social e em saúde aos indígenas, independentemente do local de residência.
